# A comparison of simplified protocols of personalized dosimetry in NEN patients treated by radioligand therapy (RLT) with [^177^Lu]Lu-DOTATATE to favor its use in clinical practice

**DOI:** 10.1007/s00259-023-06112-8

**Published:** 2023-01-23

**Authors:** Valentina Pirozzi Palmese, Laura D’Ambrosio, Francesca Di Gennaro, Costantina Maisto, Roberta de Marino, Anna Morisco, Sergio Coluccia, Piergiacomo Di Gennaro, Francesco De Lauro, Marco Raddi, Paolo Gaballo, Salvatore Tafuto, Egidio Celentano, Secondo Lastoria

**Affiliations:** 1 S.C. Medicina Nucleare E Terapia Radiometabolica, INT IRCCS Fondazione G. Pascale, Naples, Italy; 2 S.C. Epidemiologia E Biostatistica, INT IRCCS Fondazione G. Pascale, Naples, Italy; 3 S.C. Sarcomi E Tumori Rari, INT IRCCS Fondazione G. Pascale, Naples, Italy

**Keywords:** RLT, Neuroendocrine neoplasms, Dosimetry, Absorbed dose, [^177^Lu]Lu

## Abstract

**Abstract:**

The role of internal dosimetry is usually proposed for investigational purposes in patients treated by RLT, even if its application is not yet the standard method in clinical practice. This limited use is partially justified by several concomitant factors that make calculations a complex process. Therefore, simplified dosimetry protocols are required.

**Methods:**

In our study, dosimetric evaluations were performed in thirty patients with NENs who underwent RLT with [^177^Lu]Lu-DOTATATE. The reference method (M0) calculated the cumulative absorbed dose performing dosimetry after each of the four cycles. Obtained data were employed to assess the feasibility of simplified protocols: defining the dosimetry only after the first cycle (M1) and after the first and last one (M2).

**Results:**

The mean differences of the cumulative absorbed doses between M1 and M0 were – 10% for kidney, – 5% for spleen, + 34% for liver, + 13% for red marrow, and + 37% for tumor lesions. Conversely, differences lower than ± 10% were measured between M2 and M0.

**Conclusion:**

Cumulative absorbed doses obtained with the M2 protocol resembled the doses calculated by M0, while the M1 protocol overestimated the absorbed doses in all organs at risk, except for the spleen.

## Introduction

Radioligand therapy (RLT) or peptide receptor radionuclide therapy (PRRT) with labelled somatostatin analogues (SSA) is an effective and well-tolerated treatment for neuroendocrine neoplasms (NEN) of gastroentero-pancreatic tract, G1 and G2 grade, that highly express somatostatin receptors in their cell membrane. A statistically significant increase in progression-free survival for patients with advanced midgut NENs, without significant side-effects, was gained as demonstrated in the phase III Netter-1 trial with [^177^Lu]Lu-DOTATATE [1]. The approved scheme of RLT (fixed activity of 7.4 GBq/cycle for four cycles with intervals of 8 weeks) does not require dosimetry. The application of fixed activity, the lack of well-established methods to calculate absorbed doses, the time required for image acquisitions and calculation, the lack of specific trained personnel, and the discomfort to the patient for image sessions negatively affect the proper continuous use of dosimetry in clinical routine and its implementation. The direct consequence of RLT without dosimetry led to define it as a sort of “radioactive chemotherapy” rather than an internal radiotherapy with β^−^ radiation (max energy 497 keV), as defined in European Council Directive 2013/59/EURATOM definition 81 [2]. However, dosimetry is usually performed only in few specialized centers, as summarized in a recent survey made in Europe [3]. On the other hand, a significant body of literature suggested to massively adopt dosimetry to improve the knowledge and use of RLT [4, 5]. This scenario will change in European countries thanks to the adoption of the EC Directive [in Italy, 6] that definitively stated the role of dosimetry-based protocols. Thus, to increase the use of dosimetry, an unmet need is mandatory to accurately define the absorbed doses to tumors and organs at risk (OARs), and that for RLT with [^177^Lu]Lu-DOTATATE is considered bone marrow and kidneys [7].

Patients with similar clinical conditions, although receiving the same activity, may frequently present different absorbed doses to organs and lesions with related different outcomes [8, 9]. Such differences are probably correlated to the density of SS receptors, to the burden of disease, and the related blood flow supplied. Thus, the doses to tumors extremely varied inter-patients and inter-lesions.

A systematic use of dosimetry during RLT would be significantly encouraged using simplified standardized protocols. In this setting, recent works evaluated simplified dosimetric methods to favor an extended use in clinical practice [10–16]. Following this trend, our study evaluated the accuracy of streamlined dosimetric methods that use measurement performed after the first or after the first and the last cycles compared with the absorbed dose calculated after each of four cycles.

## Materials and methods

### Patients

Patient profile is summarized in Table 1. Thirty NENs patients were treated in our institute from May 2019 to September 2021 by four cycles of RLT with 7.4 GBq of [^177^Lu]Lu-DOTATATE.

**Table 1:** Patients profile at study entry

Characteristic	Value
Number of patients	30
Age (years)	
Mean	59
Range	36–84
Gender	
Male	17
Female	13
Primary tumor site	
Pancreas	15
Intestine	9
Unknown	6
Sites of metastases	
Liver	29
Lymph nodes	21
Bone	13
Other	9

Before starting the infusion of [^177^Lu]Lu-DOTATATE (30–40 min in advance) and for the next 3–4 h later, a solution of 1.5 L of amino acids was administered. [^177^Lu]Lu-DOTATATE was administered intravenously within 30 min.

### Post-treatment dosimetry protocol

The patients underwent dosimetric evaluations during each cycle. Measurements were performed by sequential quantitative SPECT/CT of the abdomen (including kidney, liver, and spleen) collected at 3, 20, and 90/120 h post infusion (p.i.). If the tumor site/s was located outside this field of view (FOV), one or more additional ones were acquired. For gamma imaging, a Discovery NM/CT 670 scanner (GE Healthcare) with an integrated bright speed multidetector CT and 3/8 in NaI(Tl) crystals was used. Parameters of acquisition and reconstruction are reported in Table 2. The image timing was chosen considering that the patients were hospitalized for 24–48 h, coming back as outpatient for the imaging session at 90/120 h p.i.

**Table 2:** SPECT/CT imaging acquisition and reconstruction parameters

System	Discovery NM/CT 670 GE Healthcare dual head SPECT/CT
Collimator	MEGP
Main energy peak	208 keV ± 10%
Scatter energy peak	178 keV ± 5%
SPECT	Number of projection: 120 Matrix: 128 × 128 Time of acquisition for view: 20/20/25 s
CT	16 slice, 120 kV Automatic regulation in mA from SCOUT Noise index: 28 Slice thickness: 3.75 mm
Image reconstruction Image correction	OSEM (4 it, 10 sub) Scatter DEW (auto) Attenuation (auto, from CT data) Reconstruction recovery (auto)
SPECT calibration factor	6.6 cps/MBq

Planar sensitivity of the hybrid SPECT/CT camera was calibrated on a semi-annual basis according to the manufacturer’s instructions using a Petri plate phantom with a known activity of [^177^Lu]lutetium (111–148 MBq); the measured calibration factor was 6.6 cps/MBq. To further verify the accuracy of calibration, an additional homemade phantom (a bottle of 1000 mL of water with 74–111 MBq of [^177^Lu]lutetium) was acquired using the same SPECT/CT protocol applied for imaging patients. The agreement between them was < 10%. The recovery curve was calculated by NEMA IEC body phantom using the methodology recommended by MIRD pamphlet No. 26 [17]. OAR volumes were calculated on the CT of the pre-therapy PET/CT study performed some days before RLT with [^68^ Ga]Ga-DOTATATE. Lesion volumes were drawn on PET images, setting a threshold value of 42% on maximum standardized uptake value (SUV) [18]. Only volumes above 4 cc (with a 0.5 recovery coefficient) were considered. The volumes of OARs, as well as of the lesions, were redrawn on the SPECT/CT images using a Q.Volumetrix MI toolkit (a tool of the workstation Xeleris™, GE Healthcare) to obtain the activity concentration [MBq/ml] in the given volume of interest (VOI). The absorbed doses were calculated according to MIRD formalism [17, 19, 20]. The activity concentrations were calculated for each time point to obtain the time-activity curves (TAC). TAC for OARs and tumors was fitted by a mono-exponential function or using the trapezoidal method [21, to determine the time-integrated activity coefficient (TIAC). Organ mass and TIAC were used as input data in OLINDA/EXM®v1.0 software to obtain absorbed dose per administered activity (AD/A). The absorbed dose within tumor was obtained by Model Sphere correcting the TIAC for recovery factor [16]. The BED value for kidney was calculated according to the LQ model, setting *α*/*β* = 2.6 Gy and ${T}_{\mathrm{rep}}$=2.8 h, as reported in the literature [22–25].

To quantify the amount of disease and to study the intra-patient variability, the liver tumor burden was assessed on SPECT/CT during the first RLT cycle, setting a threshold of 42%.

The activity in the whole body (WB) was obtained by an external gamma probe, a 2″ × 2″ NaI(Tl) detector, provided with a multichannel analyser, (Captus® 4000e Thyroid Uptake System from Capintec, INC) by serial measurements (1, 1.5, 4, 20, and 90/120 h p.i.). To cover the entire body of the patient, the probe was positioned 3 m far from the patient; each acquisition lasted 120 s, and the measurements were corrected for background counts. The effective half-live was calculated fitting the TAC with a bi-exponential function.

The activity in the blood was calculated by five samples drawn at 0.5 h (from the arm opposite the infusion site), 1.5, 6, 20, and 90/120 h post-injection, measured by a NaI(Tl) well counter (Gamma Counter Wizard^2^® 2480 from PerkinElmer Company, set at 208 keV ± 10% energy window) for 60 s. The well counter was previously calibrated with known activities of [^177^Lu]lutetium. The data were fitted with a bi-exponential function to assess the absorbed dose to red marrow as previously described [26–28]. This approach was selected to calculate the self-dose and the cross-dose component to the red marrow from blood samples and the whole body counts, respectively, with the red marrow-to-blood concentration ratio equal to 1 [28].

### Dosimetric methods

In this study, three methods were compared to evaluate the differences, if any, in the calculation of the cumulative ADs.

#### Method 0 (M0) or reference method

ADs were calculated for each cycle of RLT according to the methodology previously described using the images and external measurements.

#### Method 1 (M1)

The AD of the first cycle was calculated from direct measurements. The ADs from the second to the fourth cycles (*x*) were estimated according to Eq. 1, where ${AD}_{x}$ was obtained from ${AD}_{1}$ of the first cycle and the ratio of administered activity at each cycle (${A}_{x}$) divided by the administered activity in the first one (${A}_{1}$):

1 $${AD}_{x}={AD}_{1}\frac{{A}_{x}}{{A}_{1}}$$

The final cAD for this method was the sum of the three ${AD}_{x }$ to ${AD}_{1}$.

#### Method 2 (M2)

The Ads of the first and fourth cycles were calculated from direct measurements. The ADs for the second and third cycles were calculated using Eq. (1). The AD of the second cycle was derived by the first cycle and the AD of the third cycle from the fourth, as shown below,

$$\begin{array}{ccc}{AD}_{2}={AD}_{1}\frac{{A}_{2}}{{A}_{1}}& ;& {AD}_{3}={AD}_{4}\frac{{A}_{3}}{{A}_{4}}\end{array}$$

The final cAD was the sum of the four ${AD}_{x}$.

### Statistical analysis

The reliability of the simplified methods M1 and M2 was tested by comparing the differences between the cADs extrapolated with the reference value obtained with M0. A non-parametric Wilcoxon signed rank test for paired data was performed with a significance of 95% [29]. The null hypothesis, H0, assumes that the average of two samples is not statistically different, so the difference of means of considered values likely goes 0. Moreover, differences in the estimated cADs of M1 and M2 vs M0 were studied using Bland–Altman analysis [30] and were graphically summarized in terms of limits of agreement (LOAs), defined as

$${\mu }_{\Delta } \pm {\mathrm{z}}_{1-\mathrm{\alpha }/2}\cdot {\sigma }_{\Delta }$$

where ${\mu }_{\Delta }$ is the mean of differences, ${ z}_{1-\mathrm{\alpha }/2}$ is the $(1-{~}^{\mathrm{\alpha }}\!\left/ \!{~}_{2}\right.)$ -th quantile of a standardized Gaussian distribution, and ${\sigma }_{\Delta }$ is the standard deviation of differences; $\mathrm{\alpha }$ was chosen as 0.05, so the 95%CI of difference mean was calculated using the following equation:

$${\mu }_{\Delta } \pm {\mathrm{z}}_{1-\mathrm{\alpha }/2}\cdot \sqrt{\frac{{{\sigma }_{\Delta }}^{2}}{n}}$$

where *n* is the number of considered data, i.e. OARs, lesions in this specific case and were reported as indicators of magnitude and possible bias of the doses calculated by M1 vs M0 and M2 vs M0 [11]. A further indicator of agreement of the methods was determined by the percentage of individuals who did not fall in LOAs (termed “out-of-bound patients” − %).

Tests and graphs were carried out using the base and ggplot2 suits by R software version 4.1.3 (R Core Teams, R Foundation for Statistical Computing, Vienna, Austria).

## Results

The mean administered activity was 7.2 ± 0.4 GBq/cycle (6.3 to 7.7 GBq/cycle), and the mean cumulative activity per patient after the four cycles was 29.0 ± 0.6 GBq (27.3 to 30.0 GBq). The whole body time-activity curve showed a biexponential trend and revealed an effective half-life of 1.79 h post administration (rapid component) and a second slower component of 61.9 h, as summarized in Table 3.

**Table 3:** Bi-exponential fit parameters of fraction injection of activity [*FIA (*t*) = *A*1* exp^ − (*t*/*t*1) + *A*2*exp^ − (*t*/*t*2)] within the body of patient

*A*1	*A*2	*t*1 [*h*]	*t*2 [*h*]
0.58 ± 0.13 (0.28 to 0.79)	0.38 ± 0.14 (0.20 to 0.72)	2.58 ± 1.12 (0.14 to 6.19)	89.3 ± 26.8 (45.20 to 217.40)

Data are given as mean ± standard deviation (min to max);

*t*1 and *t*2 are equal to effective half-life divided to In(2)

### Absorbed dose calculated using M0

The dosimetric evaluations related to the 30 patients focused on the dose to kidney, spleen, liver, red marrow, and tumor lesions.

The estimated mean absorbed doses for unit of administrated activity (AD/A) over all cycles were 0.54 ± 0.15 Gy/GBq for kidney, 0.64 ± 0.32 Gy/GBq for spleen, 0.67 ± 0.81 Gy/GBq for liver, 16.0 ± 6.0 mGy/GBq for red marrow, and 4.5 ± 2.9 Gy/GBq for tumor lesions. The complete dosimetric data, AD/A for each cycle, the mean absorbed dose (AD), and the cumulative absorbed dose (cAD) are shown in Table 4.

**Table 4:** TIAC and AD/A in OARs and tumor lesions for each cycle in our set of 30 patients

	*Kidneys*	*Spleen*	*Liver*	*Tumor lesions*		*Red marrow*		
*Cycle*	AD/A [Gy/GBq]	AD/A [Gy/GBq]	AD/A [Gy/GBq]	AD/A [Gy/GBq]		Self AD/A [mGy/GBq]	Cross AD/A [mGy/GBq]	Total AD/A [mGy/GBq]
I	0.5 ± 0.2 (0.2–0.8)	0.6 ± 0.4 (0.1–1.9)	0.9 ± 0.9 (0.1–3.8)	6.2 ± 3.4 (0.3–14.6)		7.0 ± 2.0 (3.0–12.0)	11.0 ± 8.0 (3.0–40.0)	18.0 ± 8.0 (9.0–45.0)
II	0.6 ± 0.2 (0.3–1.0)	0.6 ± 0.3 (0.2–1.3)	0.7 ± 0.8 (0.1–4.0)	4.7 ± 2.4 (0.7–10.3)		7.0 ± 3.0 (3.0–20.0)	9.0 ± 5.0 (3.0–23.0)	16.0 ± 5.0 (9.0–28.0)
III	0.6 ± 0.1 (0.4–0.8)	0.7 ± 0.3 (0.1–1.5)	0.6 ± 0.8 (0.1–3.4)	3.8 ± 2.4 (0.8–10.1)		7.0 ± 4.0 (3.0–22.0)	8.0 ± 6.0 (2.0–29.0)	15.0 ± 7.0 (7.0–37.0)
IV	0.6 ± 0.1 (0.3–0.8)	0.7 ± 0.3 (0.2–1.6)	0.6 ± 0.8 (0.1–4.0)	3.4 ± 2.6 (0.7–12.3)		7.0 ± 2.0 (3.0–14.0)	8.0 ± 5.0 (2.0–22.0)	15.0 ± 5.0 (8.0–29.0)
AD [Gy]	3.9 ± 0.9 (2.4–5.6)	4.6 ± 1.9 (1.3–8.8)	4.9 ± 5.8 (0.6.–28.0)		33.0 ± 21.1 (4.8–88.6)	-	-	120.0 ± 40.0 (70.0–240.0)
cAD [Gy]	15.7 ± 3.5 (9.6–22.2)	18.5 ± 7.7 (5.3–35.0)	19.4 ± 23.1 (2.3–112.0)		131.7 ± 66.9 (22.4–267.9)	-	-	460.0 ± 170.0 (290.0–950.0)

Data are given as mean ± standard deviation (min to max)

*Three patients have undergone a splenectomy surgery

**In three patients, no blood sample had been collected for organizational reasons

TIAC changed during RLT, showing an increasing trend from cycle I to cycle IV in the kidney (mean difference =  − 0.21 ± 0.39 h, *p* < 0.01) and a decreasing one in the liver (mean difference = 5.1 ± 14.16 h, *p* < 0.01) and in the WB (mean difference = 9.37 ± 15.19 h, *p* < 0.01), but remained constant in spleen (mean difference =  − 0.18 ± 0.96 h, *p* = 0.12) and red marrow (mean difference =  − 0.10 ± 0.05 h, *p* = 0.64). The TIAC variation implied a variation of the absorbed dose (complete data set was reported in Tables 6 and 7 of Appendix).

The measured renal cAD did not exceed the dose of 23 Gy in none of the 30 patients. BED value to kidney was 16.9 ± 4.0 (10.0–24.5) Gy. In all the patients, BED was significantly lower than the limit dose of 40 Gy without risk factor (28 Gy in the presence of risk factor), enabling extra cycle/s of RLT. In detail, considering the lower limit of 28 Gy, 6 patients could receive one extra cycle, 8 patients two extra cycles, 5 patients three and 7 patients four. The measured cAD in red marrow was far below the limit dose of 2 Gy in all the patients.

In the liver, a wide range of cAD values and TIAC with related standard deviations occurred (Table 6). These findings were likely related to the degree of liver infiltration that ranged from 2 to 98% of total liver volume. A low mean TIAC value (about 0.7 h), corresponding approximately to 2.5 Gy of cAD, was measured in patients with few numbers and small in size hepatic lesions; while it increased in patients with extended hepatic infiltration (up to124 h) corresponding approximately to 112 Gy of cAD. The presence of multiple liver metastases did not allow to discriminate healthy from infiltrated hepatic parenchyma in some patients (8/30 cases).

**Fig. 1:**
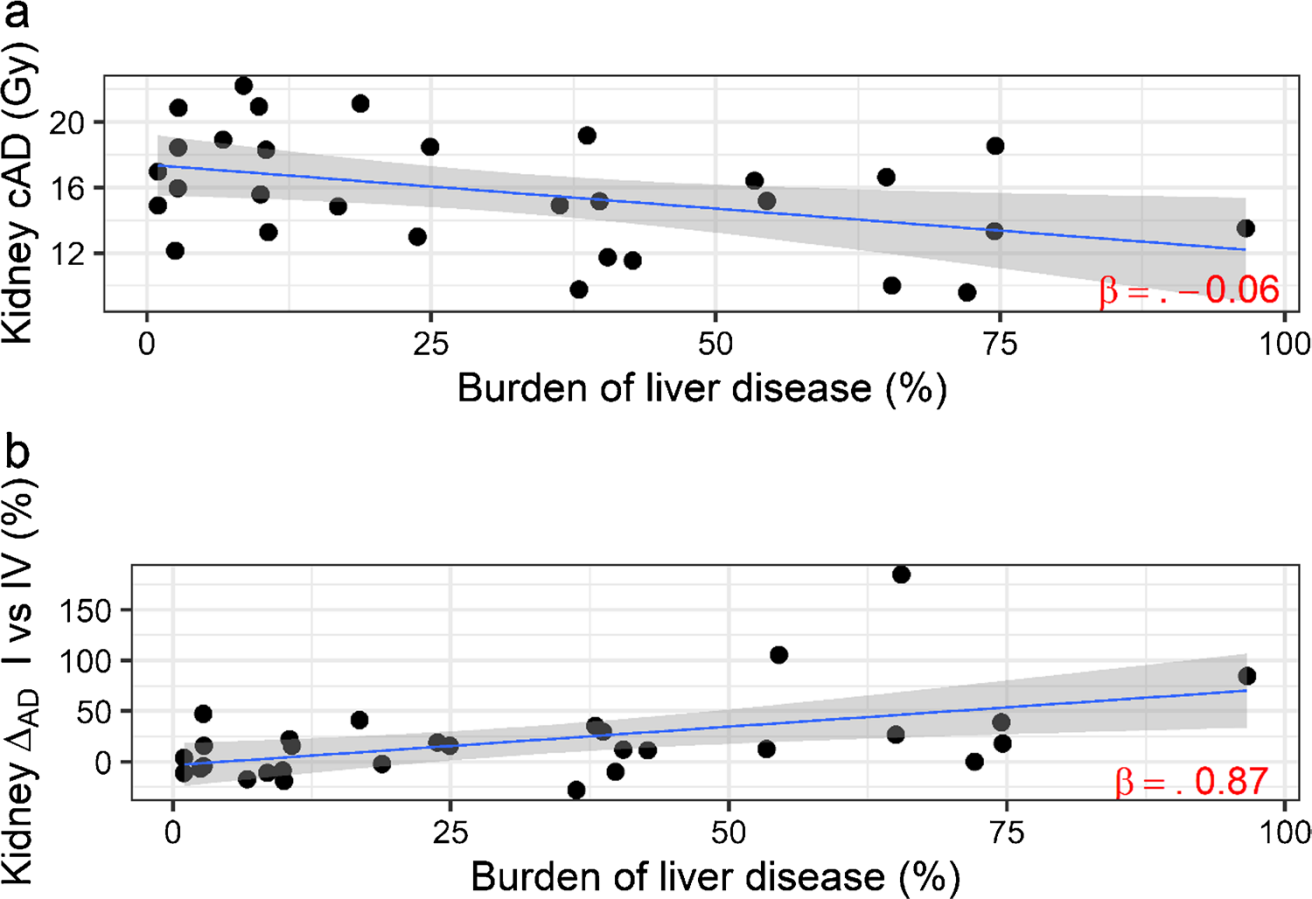
**a** Cumulative AD to kidney in function of burden of liver disease. **b** The difference of the AD to kidney at first and last cycles in function of burden of liver disease. Coefficients were adjusted for gender and weight (kg)

Figure 1a shows a non-null small linear dependence of the cumulative AD to kidney with the burden of liver disease (*β* =  − 0.06, *p* < 0.01); also, the difference of the AD to kidney at first and last cycles (Fig. 1b) is related to the burden of liver disease (*β* = 0.87, *p* < 0.01). Moreover, patients with total liver disease burden ≤ 20% had a higher risk to receive a > 20 Gy cAD to kidney (4 vs 10 compared to 0 vs 16, Fisher’s exact test, *p* = 0.04).

A wide heterogeneity of radioligand uptake occurred among lesions in the same patient and among different patients. The anatomical location influenced the degree of uptake: bone and lymph node metastases showed lower uptake than hepatic and pancreatic lesions. Similarly, significant differences were found in the uptake among cycles that were mirrored by AD variation in OARs and tumor sites.

For instance, the contribution of each cycle to cAD increased in kidney from 22% of the first cycle to 26% of the fourth cycle and in spleen from 24 to 26%.

Conversely, in other OARs, a progressive reduction of the contribution to cAD cycle by cycle was measured. In the liver, cAD decreased from 32% of the first to 20% of the fourth; in the red marrow, it decreased from 28 to 23%. In tumor lesions, a similar progressive reduction was also observed, passing from 35% of the first to 18% of the last cycle (*p* for trend = 0.02). Pancreatic lesions showed a significantly higher negative difference compared to bone lesions (*p* = 0.01), supporting the evidence that anatomical location plays a role to determine the AD to lesion.

### Cumulative absorbed dose obtained using M1 and M2

**Fig. 2:**
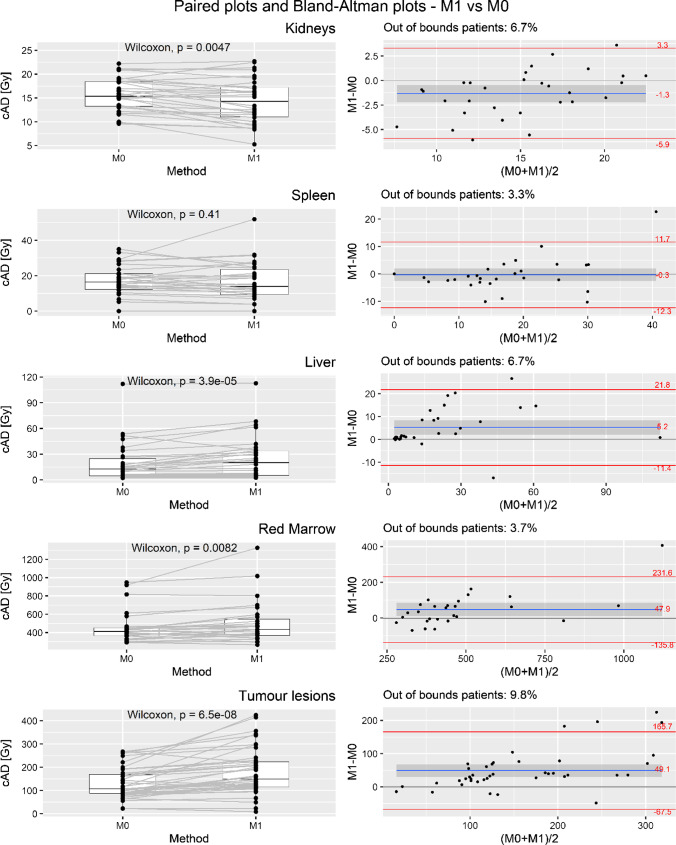
Cumulative ADs plotted for M1 and M0 comparison; to the left: paired plots and Wilcoxon rank sum test outputs; to the right: graphical tests of Bland–Altman plots, LOAs, and out-of-bound percentages; the gray band represents the 95% CI of the difference mean

**Fig. 3:**
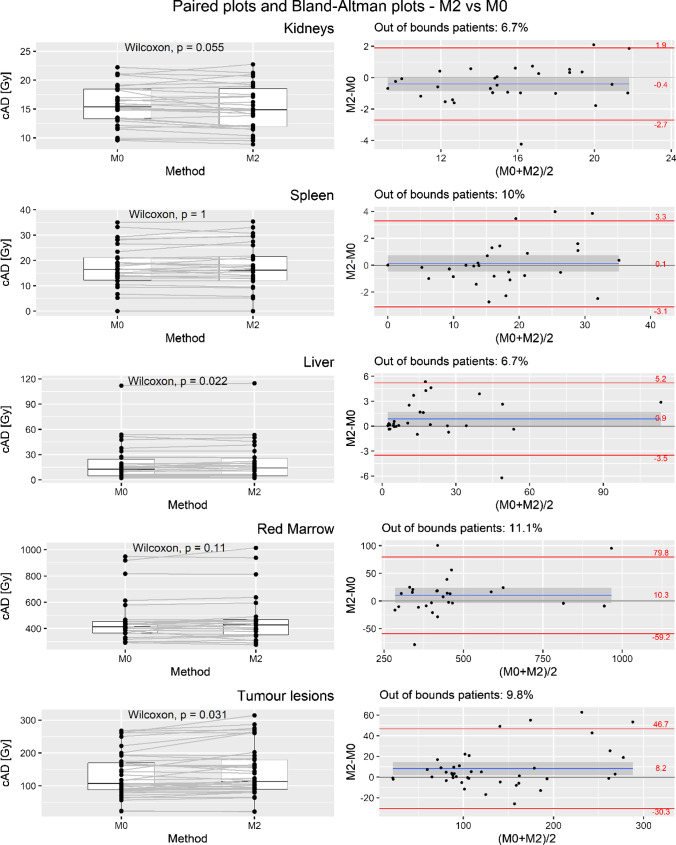
Cumulative ADs plotted for M2 and M0 comparison; to the left: paired plots and Wilcoxon rank sum test outputs; to the right: graphical tests of Bland–Altman plots, LOAs, and out-of-bound percentages; the grey band represents the 95% CI of the difference mean

Cumulative AD values for OARs and lesions obtained using methods M1 and M2 are summarized in Table 5 along with difference/s with the reference method M0 and the results of the statistical results. The cADs plotted for M1 and M2 vs M0 comparison and graphical Bland–Altman tests are reported in Figs. 2 and 3.

**Table 5:** Cumulative AD for VOI obtained with M0, M1, and M2 difference between them and *p*-values obtained from the Wilcoxon signed rank test for median differences

*Region*	M0 [Gy]	M1 [Gy]	M2 [Gy]	(M1 − M0)/M0 [%]	(M2 − M0)/M0 [%]	*p value* (M1 vs M0)	*p value* (M2 vs M0)
Kidney	15.7 ± 3.5 (9.6–22.2)	14.4 ± 4.6 (5.3–22.7)	15.3 ± 3.8 (8.9–22.7)	− 9.7 ± 16.0 (− 47.3 to 19.0)	− 2.8 ± 7.0 (− 23.2 to 11.1)	< 0.01	0.055
Spleen	18.5 ± 7.7 (5.3–35.0)	18.1 ± 10.5 (3.9–51.9)	18.6 ± 8.2 (5.1–35.4)	− 4.9 ± 29.3 (− 52.6 to 77.7)	− 0.3 ± 8.8 (− 16.3 to 19.5)	0.41	1
Liver	19.4 ± 23.1 (2.3–112.0)	24.6 ± 25.3 (2.6–112.8)	21.5 ± 23.7 (2.3–114.8)	33.6 ± 40.6 (− 32.5128.8)	6.3 ± 12.5 (− 12.3 to 35.8)	< 0.01	< 0.01
Red Marrow	0.46 ± 0.17 (0.29–0.95)	0.51 ± 0.23 (0.27–1.32)	0.47 ± 0.18 (0.28–1.01)	12.5 ± 18.5 (− 27.1 to 49.2)	2.6 ± 7.8 (− 9.5 to 21.6)	< 0.01	0.11
Tumor lesions	131.7 ± 66.9 (22.4–267.9)	180.7 ± 99.6 (9.0–424.7)	139.9 ± 74.6 (21.2–314.5)	37.4 ± 44.2 (− 61.3 to 157.1)	5.8 ± 13.2 (− 15.2 to 42.2)	< 0.01	< 0.01

Data are given as mean ± SD (min to max)

M1 underestimated cAD to the kidney with a mean difference ranging from − 9.7 ± 16.0% (with a maximal underestimation of − 47.3% and a maximal overestimation of up 19.0%); the *p-*value was < 0.01. Similarly, for red marrow, the difference between M0 and M1 was 12.5 ± 18.5% (with an underestimation of up to − 27.1% and an overestimation of up 49.2%) and the *p*-value was < 0.01.

Regarding the M1 vs M0 comparison, LOAs (Gy) were − 5.9 and 3.3 Gy for kidney, − 12.3 and 11.7 Gy for spleen, − 11.4 and 21.8 Gy for liver, − 135.8 and 231.6 mGy for red marrow, and − 67.5 and 165.7 Gy for tumor lesions. Out-of-bound patients’ percentage was 6.7% for kidney, 3.3% for spleen, 6.7% for liver, 3.7% for red marrow, and 9.8% for tumor lesions. The empirical value of 5% (corresponding to the probability of moving away from the mean value by more than almost 2 times the standard deviation of a Gaussian distribution) was not exceeded by spleen and red marrow (see Fig. 2, second column).

The mean difference applying M2 vs M0 was not significant in kidney, spleen, and red marrow. In the kidney, the mean difference was − 2.8 ± 7.0% (with an underestimation of up to − 23.2% and an overestimation of up to 11.1%) and the *p*-value was 0.055. For red marrow, the mean difference was 2.6 ± 7.8% (with an underestimation of up to − 9.5% and an overestimation of up 21.6%) and the *p*-value was 0.11.

Comparing the M2 method vs M0 (see Fig. 3, second column), LOAs were − 2.7 and 1.9 Gy for kidney, − 3.1 and 3.3 Gy for spleen, − 3.5 and 5.2 Gy for liver, − 69.2 and 79.8 mGy for red marrow, and − 30.3 and 46.7 Gy for tumor lesions. Out-of-bound patients’ percentage was 6.7% for kidney, 10.0% for spleen, 6.7% for liver, 11.1% for red marrow, and 9.8% for tumor lesions, so all the regions were beyond the empirical value of 5%.

The value of 0, which represents a consideration of non-bias between the two methods (M1 and M2 vs M0), was not contained in the 95% CI of difference means for kidney [− 2.2, − 0.5], liver [2.2, 8.3], red marrow [12.5, 83.2], and tumor lesions [30.9, 67.3] in M1 vs M0 and for liver [0.1, 1.7] and tumor lesions [2.2, 14.2] comparing M2 with M0. All the statistical results are summarized in Table 5.

## Discussion

RLT plays a pivotal role in the treatment of patients with NENs and, in the near future, it will be extended to other solid tumors (i.e., PSMA inhibitors in prostate carcinoma). The assessment of an accurate dosimetry to OARs and tumor lesions could establish a more balanced comparison between potential benefit and toxicity [31, 32].

To adopt dosimetry in clinical routine is mandatory to simplify the collection of required data and to harmonize the protocols.

In this setting, our study evaluated the role of simplified methods tested in a series of 30 patients with NEN. The ADs to OARs measured in these patients agreed with previously published data [33–35] (Table 8 in Appendix). None of the patients exceeded the limit dose for kidney and red marrow. The renal dose of 23 Gy was not reached, as well as the 28–40 Gy BED [23]. This result offers the opportunity to consider additional cycles of RLT to the standard four, as well as reported in other papers [36, 37]. In details, only 4 patients (14%) should stop therapy, while 26 (86%) might receive additional (from 1 up to 4) cycles of RLT. Therefore, the calculation of cumulative kidney dose plays a pivotal role in decision-making of a possible retreatment in case of persistence of positivity to SSA after the therapy [38, 39].

No significant hematological toxicity (G3) correlation with therapy was observed after 6/12 months; 12 patients showed short-term, reversible toxicity (G1 and G2). The assessment of absorbed dose in the red marrow was obtained using the same protocol adopted in the NETTER-1 trial that is probably not the gold standard for RLT but widely used for radioimmunotherapy and iodine therapy, waiting for well-established images-based method.

However, the AD to the OARs changes during RLT, being influenced not only by organ uptake but also by tumor uptake. For instance, the evident reduction of uptake within tumor/s during the four cycles of RLT makes available a greater amount of radioligand for the uptake in the OARs. In addition, we found that the liver tumor burden is inversely correlated to the kidney absorbed dose (Fig. 1). In patients with low hepatic metastatization (< 20% of total liver volume) a cAD > 20 Gy was measured.

In this series, the ADs in tumors drastically decreased after the first cycle (Table 4). This result has been already published for patients with pancreatic NENs, and it has been correlated with progressive reduction of neoplastic vasculature [9, 32]. The progressive decline of effectiveness of RLT from the first to the last cycles might suggest to reevaluate the current strategy. For instance, an increase of the injected activity at first treatment might be considered to take advantage from the highest uptake in tumor lesions.

Once utility of dosimetry has been demonstrated [36–39], the goal is to define a suitable method that might represent a win–win situation for patients and nuclear medicine resources by reducing time and the number of imaging sessions.

Different strategies have been proposed to reduce the number of patient return and post-treatment studies after each cycle of RLT. Hanscheid et al. [10] proposed to acquire SPECT/CT at a single time-point (96 h p.i.) to reliably assess AD with an error ≤ 10%. Willowson et al. [12] suggested to calculate kidney AD using a single SPECT/CT associated with a theoretical renal clearance half-life of the peptide. Chicheportiche et al. [13] assessed the feasibility of a two-time point dosimetry protocol with SPECT/CT studies acquired after the first cycle of treatment and at early single-time point after the following ones. In another work, the same author [14] showed that dosimetric calculations, using a multiple linear regression model with a single SPECT/CT study, were in agreement with standard imaging protocol by reducing treatment-related expenses and scanner/staff time.

Our study proposes to perform dosimetry after one or two cycles and compares these simplified methods with data obtained after each cycle (reference method, M0) of RLT. The choice of the fourth cycle for the M2 method rather than the third one was based on the evidence that the latter better considered the major variability in the radiopharmaceutical uptake.

The extrapolation of ADs, derived from the dosimetric evaluations performed only at the first cycle (M1), showed a weak correlation with ADs calculated by M0. A mean cAD variation of about − 10% for kidney, − 5% for spleen, and 13% for red marrow was found. For the liver and tumor lesions, M1 overestimated the AD of approximately 34 and 37%, respectively. This overestimation was a consequence of the reduced uptake after the other three cycles.

All these values decreased when dosimetry was calculated by M2: − 3% for kidney, − 0.3% for spleen, 6% for liver, 3% for red marrow, and 6% for tumor lesions. The better correlation with M0 seems to be justified by the identification of the changes in the uptake (namely, tumors) that occurred during the RLT.

The results of statistical tests indicated that the cumulative AD difference obtained with M0 and M1 methods was significant for all the regions investigated, except the spleen. On the other hand, the difference of cumulative ADs obtained with M0 and M2 methods was not statistically significant in organs without lesions (kidney, spleen, and red marrow). In summary, as reported in 95% CI of mean differences, M1 underestimated the dose to the kidney and overestimated the dose to liver, red marrow, and tumor lesions. The M2 method permitted to obtain cADs with a better accuracy.

Moreover, the evaluations done with M2 encouraged to adopt this simplified dosimetry protocol for all new patients recruited into RLT with [^177^Lu]Lu-DOTATATE. This choice represents a good compromise between costs and benefits.

## Conclusions

Performing dosimetry during RLT using a simplified method is feasible and easy to implement in clinical routine. According to our experience, cumulative absorbed doses extrapolated from the first and fourth cycles are in good agreement with dosimetry done at each cycle. These results can improve patient comfort and spare scanners and technologists’ camera time, enabling a reduction in treatment expenses without compromising patient safety. Moreover, simplified methods resulted in being able to improve cost/benefit ratio and simultaneously assuring the same accuracy.

## Appendix

**Table 6:** TIAC and AD/A in OARs and tumor lesions for each cycle in *our set of* 30 patients

	*Kidneys*		*Spleen*		*Liver*		*Tumor lesions*	
*Cycle*	TIAC [h]	AD/A [Gy/GBq]	TIAC [h]	AD/A [Gy/GBq]	TIAC [h]	AD/A [Gy/GBq]	TIAC [h]	AD/A [Gy/GBq]
I	1.7 ± 0.6 (0.3–3.0)	0.5 ± 0.2 (0.2–0.8)	1.6 ± 1.1 (0.1–4.9)	0.6 ± 0.4 (0.1–1.9)	20.1 ± 24.9 (1.1–86.1)	0.9 ± 0.9 (0.1–3.8)	3.0 ± 5.4 (0.2–24.7)	6.2 ± 3.4 (0.3–14.6)
II	1.9 ± 0.6 (0.6–3.3)	0.6 ± 0.2 (0.3–1.0)	1.6 ± 1.0 (0.1–4.3)	0.6 ± 0.3 (0.2–1.3)	17.6 ± 19.7 (0.7–139.5)	0.7 ± 0.8 (0.1–4.0)	2.5 ± 4.9 (0.1–21.5)	4.7 ± 2.4 (0.7–10.3)
III	1.9 ± 0.5 (0.8–2.7)	0.6 ± 0.1 (0.4–0.8)	1.7 ± 1.0 (0.1–4.5)	0.7 ± 0.3 (0.1–1.5)	16.2 ± 29.5 (0.6–137.6)	0.6 ± 0.8 (0.1–3.4)	2.3 ± 5.4 (0.1–28.6)	3.8 ± 2.4 (0.8–10.1)
IV	1.9 ± 0.4 (1.0–2.7)	0.6 ± 0.1 (0.3–0.8)	1.7 ± 1.1 (0.2–4.9)	0.7 ± 0.3 (0.2–1.6)	14.9 ± 28.9 (0.7–134.6)	0.6 ± 0.8 (0.1–4.0)	2.1 ± 5.4 (0.1–31.6)	3.4 ± 2.6 (0.7–12.3)
AD [Gy]	3.9 ± 0.9 (2.4–5.6)		4.6 ± 1.9 (1.3–8.8)		4.9 ± 5.8 (0.6.–28.0)		33.0 ± 21.1 (4.8–88.6)	
cAD [Gy]	15.7 ± 3.5 (9.6–22.2)		18.5 ± 7.7 (5.3–35.0)		19.4 ± 23.1 (2.3–112.0)		131.7 ± 66.9 (22.4–267.9)	

Data are given as mean ± standard deviation (min to max)

*Three patients have been undergone a splenectomy surgery

**Three patients no blood sample have been collected for organizational reasons

**Table 7:** TIAC in the red marrow, in the whole body, the self, cross, and total AD/A in the red marrow for each cycle in 30 patients

*RedMarrow*					
*Cycle*	RM* TIAC [h]	WB** TIAC [h]	Self AD/A [mGy/GBq]	Cross AD/A [mGy/GBq]	Total AD/A [mGy/GBq]
I	0.18 ± 0.07 (0.07–0.30)	37 ± 28 (7–135)	7.0 ± 2.0 (3.0–12.0)	11.0 ± 8.0 (3.0–40.0)	18.0 ± 8.0 (9.0–45.0)
II	0.19 ± 0.09 (0.08–0.48)	30 ± 18 (11–86)	7.0 ± 3.0 (3.0–20.0)	9.0 ± 5.0 (3.0–23.0)	16.0 ± 5.0 (9.0–28.0)
III	0.19 ± 0.12 (0.07–0.73)	26 ± 19 (7–99)	7.0 ± 4.0 (3.0–22.0)	8.0 ± 6.0 (2.0–29.0)	15.0 ± 7.0 (7.0–37.0)
IV	0.19 ± 0.06 (0.06–0.29)	26 ± 17 (6–79)	7.0 ± 2.0 (3.0–14.0)	8.0 ± 5.0 (2.0–22.0)	15.0 ± 5.0 (8.0–29.0)
AD [mGy]	-	-	-	-	120.0 ± 40.0 (70.0–240.0)
cAD [mGy]	-	-	-	-	460.0 ± 170.0 (290.0–950.0)

Data are given as mean ± SD (min to max)

RM*, red marrow.

WB**, whole body

**Table 8:** Radiation dose reported in literature for kidney and red marrow. Values are given as absorbed dose per unit activity or absorbed dose. Mean ± SD or median (range)

Organ	Radiation dose	Reference
Kidney	0.54 ± 0.15 Gy/GBq 3.9 ± 0.9 Gy/cycle 15.7 ± 3.5 Gy *on four cycle	Present study
	0.43 ± 0.13 Gy/GBq	Walrand et al. [33]
	4.5 (1.8–8.9) Gy right 4.4 (1.7–9.8) Gy left *At first cycle	Sandstrom et al. [34]
	0.88 ± 0.19 Gy/GBq 0.62 (0.45–17.74) Gy/GBq 0.9 ± 0.3 Gy/GBq (0.32–1.67) Gy/GBq	Bodei et al. [35]
Red Marrow	0.016 ± 0.006 Gy/GBq 0.12 ± 0.07 Gy/cycle 0.46 ± 0.17 Gy *on four cycle	Present study
	0.02 (0.01–0.03) Gy/GBq	Walrand et al. [33]
	0.136 (0.056–0.507) Gy 0.07–0.51 Gy *at first cycle	Sandstrom et al. [34]
	0.07 ± 0.01 Gy/GBq 0.04 (0.02–0.06) Gy/GBq 0.04 ± 0.02 Gy/GBq 0.02 ± 0.03 Gy/GBq	Bodei et al. [35]

## Data Availability

The datasets generated and analyzed during the current study are available from the corresponding author upon reasonable request. Raw data link: https://zenodo.org/record/6966680#.Yuzs7nZBxaQ.
